# Supplementary material for: Immune Durability and Breakthrough Infections 15 Months After SARS-CoV-2 Boosters in People over 65: The IMMERSION Study
Source: Vaccines (Basel). 2025 Jul 9;13(7):738. doi: 10.3390/vaccines13070738 (PMC12298634; doi:10.3390/vaccines13070738)
Supplement: Supplementary file 1 [file vaccines-13-00738-s001.zip › vaccines-3691998-supplementary.pdf]

Supplementary Tables

Table S1. Descriptive study population of the baseline sub-cohort, with the serological response, infection and vaccination status, and sociodemographic and clinical characteristics.

	Baseline (N=86)	3-month-visit (N=86)	9-month-visit (N=77)	15-month-visit (N=76)
**Sociodemographic characteristics**				
Age, years, *median* [Q1;Q3]	69.0 [66.0;74.8]	69.0 [66.0;74.8]	69.0 [66.0;74.0]	69.0 [66.0;74.0]
Age, years (categorical), *n* (%)				
63-74	64 (74.4)	64 (74.4)	58 (75.3)	58 (76.3)
75-84	18 (20.9)	18 (20.9)	16 (20.8)	15 (19.7)
>84	4 (4.7)	4 (4.7)	3 (3.9)	3 (4.0)
Sex, female, *n* (%)	52 (60.5)	52 (60.5)	46 (59.7)	45 (59.2)
**Health problems** [1]				
Number of diseases, *n* (%)				
0	2 (2.4)	2 (2.4)	2 (2.7)	2 (2.7)
1-2	12 (14.3)	12 (14.3)	11 (14.7)	11 (14.9)
3-5	17 (20.2)	17 (20.2)	15 (20.0)	17 (23.0)
6-9	22 (26.2)	22 (26.2)	21 (28.0)	20 (27.0)
>9	31 (36.9)	31 (36.9)	26 (34.7)	24 (32.4)
**Vaccination status**				
Number of vaccine doses, *n* (%)				
2	86 (100)	.	.	.
3	.	86 (100)	77 (100)	40 (52.6)
4	.	.	.	36 (47.4)
Vaccination strategy [2], *n* (%)				
Heterologous	46 (53.5)	46 (53.5)	42 (54.5)	42 (55.3)
Homologous	40 (46.5)	40 (46.5)	35 (45.5)	34 (44.7)
**SARS-CoV-2 infection**				
Infection status, *n* (%)				
Uninfected	64 (74.4)	56 (65.1)	31 (40.2)	23 (30.3)
Infected	22 (25.6)	21 (24.4)	18 (23.4)	17 (22.4)
Reinfected	0 (0)	1 (1.2)	2 (2.6)	3 (3.9)
Breakthrough	0 (0)	8 (9.3)	26 (33.8)	33 (43.4)
**Humoral immune response**				
IgG(N), Log_{10} (BAU/mL), *mean* (SD)	0.46 (0.91)	0.57 (0.78)	1.07 (0.92)	1.28 (0.82)
IgG(S), Log_{10} (BAU/mL), *mean* (SD)	2.22 (0.73)	3.42 (0.45)	3.47 (0.59)	3.54 (0.60)
Neutralization capacity, Log_{10} (reciprocal dilution), *mean* (SD)	1.96 (0.49) *(n=79)*	2.74 (0.62) *(n=86)*	2.69 (0.62) *(n=77)*	2.88 (0.72) *(n=75)*

[1] Health problems were not available for two individuals. [2] The vaccination of subjects who received three doses of an mRNA vaccine (Pfizer-BioNTech or Moderna) is counted as a homologous vaccination, while the vaccination of subjects who received at least one dose of a viral vector vaccine (AstraZeneca or Janssen) is counted as a heterologous vaccination.

Table S2. Descriptive study population regarding the number of vaccine doses at 15 months, with the serological response, infection and vaccination status, and sociodemographic and clinical characteristics.

	3 vaccine doses (N=73)	4 vaccine doses (N=160)
**Sociodemographic characteristics**		
Age, years, *median* [Q1;Q3]	72.0 [67.0;76.0]	76.0 [72.0;82.0]
Age, years (categorical), *n* (%)		
63-74	49 (67.1)	60 (37.5)
75-84	20 (27.4)	77 (48.1)
>84	4 (5.5)	23 (14.4)
Sex, female, *n* (%)	42 (57.5)	81 (50.6)
**Health problems** [1]		
Number of diseases, *n* (%)		
0	1 (1.4)	2 (1.3)
1-2	7 (10.0)	19 (12.1)
3-5	10 (14.3)	32 (20.4)
6-9	28 (40.0)	39 (24.8)
>9	24 (34.3)	65 (41.4)
**Vaccination status**		
Vaccination strategy [2], *n* (%)		
Heterologous	29 (39.7)	14 (8.8)
Homologous	44 (60.3)	146 (91.2)
Time from fourth dose to 15-month-visit, days, *median* [Q1;Q3]	.	89 [70;108]
**SARS-CoV-2 infection**		
Infection status, *n* (%)		
Uninfected	17 (23.3)	36 (22.5)
Infected	14 (19.2)	52 (32.5)
Reinfected	3 (4.1)	2 (1.2)
Breakthrough	39 (53.4)	70 (43.8)
**Humoral immune response**		
IgG(N), Log_{10} (BAU/mL), *mean* (SD)	1.41 (0.84)	3.92 (0.47)
IgG(S), Log_{10} (BAU/mL), *mean* (SD)	3.41 (0.72)	3.92 (0.47)
Neutralization capacity [3], Log_{10} (reciprocal dilution), *mean* (SD)	2.69 (0.62)	2.88 (0.72)
**Cellular immune response** [4]		
Cellular response, pg/mL, *median* [Q1;Q3]	78.4 [13.3;120]	87.7 [41.0;163]
Cellular status, *n* (%)		
Positive	8 (72.7)	13 (100)
Negative	0 (0)	0 (0)
Undetermined	3 (27.3)	0 (0)
Not valuable	0 (0)	0 (0)

[1] Health problems were not available for six individuals. [2] The vaccination of subjects who received three doses of an mRNA vaccine (Pfizer-BioNTech or Moderna) is counted as a homologous vaccination, while the vaccination of subjects who received at least one dose of a viral vector vaccine (AstraZeneca or Janssen) is counted as a heterologous vaccination. [3] Neutralizing activity was tested in a total of 179 individuals (64 with three doses). [4] The cellular study was performed in a total of 24 individuals.

Table S3. Descriptive study population regarding the participants' sex, with the serological response, infection and vaccination status, and sociodemographic and clinical characteristics.

	Baseline (N=86)			3-month-visit (N=287)			9-month-visit (N=249)			15-month-visit (N=233)		
**Age groups**	63-74 *years* (n=64)	75-84 *years* (n=18)	>84 *years* (n=4)	63-74 *years* (n=120)	75-84 *years* (n=121)	>84 *years* (n=46)	63-74 *years* (n=110)	75-84 *years* (n=101)	>84 *years* (n=38)	63-74 *years* (n=109)	75-84 *years* (n=97)	>84 *years* (n=27)
**Sociodemographic characteristics**												
Sex, female, *n* (%)	37 (57.8)	13 (72.2)	2 (50.0)	71 (59.2)	60 (49.6)	21 (45.7)	65 (59.1)	51 (50.5)	18 (47.7)	64 (58.7)	49 (50.5)	10 (37.0)
**Health problems [1]**												
Number of diseases, *n* (%)												
0	2 (3.2)	0 (0)	0 (0)	2 (1.7)	1 (0.8)	0 (0)	2 (1.8)	1 (1.0)	0 (0)	2 (1.9)	1 (1.1)	0 (0)
1-2	9 (14.3)	3 (16.7)	0 (0)	17 (14.4)	12 (10.2)	1 (2.2)	15 (13.9)	11 (11.2)	1 (2.7)	15 (14.0)	10 (10.6)	1 (3.8)
3-5	16 (25.4)	1 (5.5)	0 (0)	30 (25.4)	14 (11.9)	9 (20.0)	26 (24.1)	10 (10.2)	7 (18.9)	27 (25.2)	10 (10.6)	5 (19.2)
6-9	17 (27.0)	4 (22.2)	1 (33.3)	32 (27.1)	36 (30.5)	14 (31.1)	31 (28.7)	31 (31.6)	11 (29.7)	30 (28.0)	29 (30.9)	8 (30.8)
>9	19 (30.1)	10 (55.6)	2 (66.7)	37 (31.4)	55 (46.6)	21 (46.7)	34 (31.5)	45 (45.9)	18 (48.6)	33 (30.8)	44 (46.8)	12 (46.2)
**Vaccination status**												
Number of vaccine doses, *n* (%)												
2	64 (100)	18 (100)	4 (100)	.	.	.	.	.	.	.	.	.
3	.	.	.	120 (100)	121 (100)	46 (100)	110 (100)	101 (100)	38 (100)	49 (45.0)	20 (20.6)	4 (14.8)
4	.	.	.	.	.	.	.	.	.	60 (55.0)	77 (79.4)	23 (85.2)
Vaccination strategy [2], *n* (%)												
Heterologous	46 (71.9)	0 (0)	0 (0)	47 (39.2)	0 (0)	0 (0)	43 (39.1)	0 (0)	0 (0)	43 (39.4)	0 (0)	0 (0)
Homologous	18 (28.1)	18 (100)	4 (100)	73 (60.8)	121 (100)	46 (100)	67 (60.9)	101 (100)	38 (100)	66 (60.6)	97 (100)	27 (100)
**SARS-CoV-2 infection**												
Infection status, *n* (%)												
Uninfected	46 (71.9)	15 (83.3)	3 (75.0)	82 (68.3)	81 (66.9)	28 (60.9)	38 (34.5)	36 (35.6)	11 (28.9)	23 (21.1)	25 (25.8)	5 (18.5)
Infected	18 (28.1)	3 (16.7)	1 (25.0)	30 (25.0)	39 (32.2)	18 (39.1)	28 (25.5)	30 (29.7)	13 (34.2)	27 (24.8)	29 (29.9)	10 (37.0)
Reinfected	0 (0)	0 (0)	0 (0)	1 (0.8)	0 (0)	0 (0)	2 (1.8)	2 (2.0)	2 (5.3)	3 (2.7)	1 (1.0)	1 (3.7)
Breakthrough	0 (0)	0 (0)	0 (0)	7 (5.8)	1 (0.8)	0 (0)	42 (38.2)	33 (32.7)	12 (31.6)	56 (51.4)	42 (43.3)	11 (40.7)
**Serological response**												
IgG(N), Log_{10} (BAU/mL), *mean* (SD)	0.51 (0.91)	0.23 (0.92)	0.78 (0.76)	0.61 (0.72)	0.71 (0.63)	0.81 (0.77)	1.19 (0.84)	1.14 (0.91)	1.22 (0.92)	1.40 (0.77)	1.29 (0.81)	1.29 (0.87)
IgG(S), Log_{10} (BAU/mL), *mean* (SD)	2.24 (0.70)	2.03 (0.69)	2.79 (0.48)	3.39 (0.43)	3.41 (0.43)	3.44 (0.48)	3.53 (0.58)	3.46 (0.58)	3.56 (0.67)	3.72 (0.60)	3.80 (0.57)	3.81 (0.76)

Neutralization capacity [3], Log_{10} (reciprocal dilution), *mean* (SD)	1.98 (0.48) *(n=60)*	1.78 (0.02) *(n=16)*	2.57 (1.38) *(n=3)*	2.75 (0.62) *(n=107)*	2.63 (0.68) *(n=72)*	2.82 (0.75) *(n=46)*	2.74 (0.66) *(n=97)*	2.79 (0.74) *(n=61)*	2.79 (0.75) *(n=38)*	2.93 (0.69) *(n=94)*	2.94 (0.70) *(n=58)*	2.86 (0.72) *(n=27)*
Cellular response [4], pg/mL, *median* [Q1;Q3]	.	.	.	74.5 [41.2;156] *(n=22)*	90.0 [26.3;362] *(n=7)*	7.20 [7.20;7.20] *(n=1)*	88.3 [29.6;154] *(n=18)*	159 [40.4;257] *(n=6)*	.	84.9 [33.9;123] *(n=18)*	81.0 [37.7;152] *(n=6)*	.

[1] Health problems were not available for six individuals (two from the baseline subgroup). [2] The vaccination of subjects who received three doses of an mRNA vaccine (Pfizer-BioNTech or Moderna) is counted as a homologous vaccination, while the vaccination of subjects who received at least one dose of a viral vector vaccine (AstraZeneca or Janssen) is counted as a heterologous vaccination. [3] Neutralizing activity was tested in a total of 225 individuals (79 from the baseline subgroup). [4] The cellular study was not performed at the baseline visit and only performed in a total of 31 individuals in following visits.

Table S4. Infection status and diagnosis, before and 3, 9, and 15 months after the administration of the third dose of the SARS-CoV-2 vaccine.

	**Baseline**				**3-month-visit**				**9-month-visit**				**15-month-visit**			
**Infection group**	Uninfected	Infected	Reinfected	Break-through	Uninfected	Infected	Reinfected	Break-through	Uninfected	Infected	Reinfected	Break-through	Uninfected	Infected	Reinfected	Break-through
**Diagnosis [1], *n***																
RT-PCR	.	7	0	0	.	20	0	0	.	16	2	4	.	15	2	3
Ag-RT	.	5	0	0	.	15	1	5	.	14	4	16	.	13	3	17
Serology	.	10	0	0	.	52	0	3	.	41	0	67	.	38	0	89
**Total**	64	22	0	0	191	87	1	8	85	71	6	87	53	66	5	109

[1] Subjects with a positive test (RT-PCR or Ag-RT) have been included in the groups diagnosed with RT-PCR or Ag-RT, respectively. Subjects with no test but positive serology have been included in the group diagnosed with IgG(N). If the subject has a positive test and positive serology, the test has been taken into account. If the infection status does not change in following-up visits, the diagnostic type remains the same as the previous visit.

Table S5. Logistic regression analysis of post-vaccine infection in previously infected and previously uninfected populations. Analysis of the risk of a new infection between 3 and 9 months after the administration of the third dose of the SARS-CoV-2 vaccine in previously infected (A) and previously uninfected (B) subjects, based on the levels of IgG(S) and neutralizing antibodies, and adjusted by age and sex.

	A			B		
		**Infected (N=70)**			**Uninfected (N=127)**	
	OR	95%CI	*p-value*	OR	95%CI	*p-value*
IgG(S) *	1.089	[0.049;43.61]	*0.959*	1.372	[0.386;4.951]	*0.625*
Neutralizing antibodies *	0.524	[0.040;5.385]	*0.590*	0.665	[0.284;1.516]	*0.336*
Age (*Ref*. 63-74 years)						
75-84	1.540	[0.048;46.30]	*0.781*	0.603	[0.258;1.379]	*0.235*
>84	6.304	[0.499;157.1]	*0.168*	1.060	[0.397;2.847]	*0.907*
Sex (*Ref*. female)	0.207	[0.010;1.799]	*0.192*	1.028	[0.490;2.149]	*0.942*

* log transformation.
